# Feasibility of Use of a Smart Speaker to Administer Snellen Visual Acuity Examinations in a Clinical Setting

**DOI:** 10.1001/jamanetworkopen.2020.13908

**Published:** 2020-08-19

**Authors:** Haroon O. Ismail, Akini R. Moses, Matthew Tadrus, Essa A. Mohamed, Leslie S. Jones

**Affiliations:** 1 Department of Ophthalmology, Howard University College of Medicine, Washington, DC; 2 Department of Cardiovascular Medicine, Mayo Clinic, Rochester, Minnesota

## Abstract

This diagnostic study investigates whether there are clinically significant differences between Snellen visual acuity examinations performed by a human vs a smart speaker.

## Introduction

Despite the growing interest in and widespread adoption of smart speakers in many industries, these devices have not been evaluated for use in clinical settings.^1,2^ In this study, we assessed the feasibility, reliability, precision, and accuracy of using the most ubiquitous smart speaker, Amazon Alexa,^3^ to perform Snellen visual acuity examinations autonomously in a clinical setting.

## Methods

This diagnostic study was conducted at the Howard University (HU) Department of Ophthalmology from April 11, 2018, to May 18, 2018, and was approved by the HU institutional review board. Participants were recruited through convenience sampling at the HU College of Medicine and the HU Ophthalmology Clinic and included those who met the eligibility criteria (eAppendix in the Supplement). Participation was strictly voluntary, written informed consent was obtained, and no compensation was provided. This study followed the Transparent Reporting of Evaluations With Nonrandomized Designs (TREND) reporting guidelines.

Each participant served as their control and underwent 2 versions of the Snellen visual acuity examination: the standard computerized examination administered by trained personnel (the control), and the examination administered by the smart speaker (the treatment). After the completion of both versions of the examination, all deidentified participant results were then sent to a cloud database for later review. All reported values are for both eyes (Table).

**Table.** Results From the Assessment of BCVA in the Smart Speaker and Human Administrations of the Snellen Visual Acuity Examination

Statistical calculations	Eye		
	Right	Left	Both
Sample size, No. of participants	65	65	68
Smart speaker BCVA LogMAR, mean (SEM)	0.112 (0.020)	0.108 (0.018)	0.063 (0.014)
Human BCVA LogMAR, mean (SEM)	0.090 (0.018)	0.079 (0.016)	0.049 (0.013)
*P* value for human vs smart speaker	.05	.01	.04
Difference between smart speaker and human BCVA LogMAR, mean (SEM) [95% CI]	0.022 (0.002) [0.000 to 0.043]	0.029 (0.002) [0.007 to 0.051]	0.014 (0.001) [0.007 to 0.051]
Reliability, Cronbach α	0.914	0.887	0.928
Concordance correlation coefficient (95% CI)	0.8340 (0.7460 to 0.8934)	0.7799 (0.6680 to 0.8573)	0.8585 (0.7837 to 0.9088)
Pearson ρ (precision)	0.8502	0.8024	0.8722
Bias correction factor C_b_ (accuracy)	0.9810	0.9720	0.9843
Bland-Altman limit, mean difference (95% CI)	−0.02 (−0.19 to 0.15)	−0.03 (−0.20 to 0.15)	−0.01 (−0.13 to 0.10)

Abbreviations: BCVA, best corrected visual acuity; LogMAR, logarithm of the minimum angle of resolution.

The concordance correlation coefficient (CCC) and a Bland-Altman plot were used to assess agreement between the tests administered by the smart speaker and by personnel. The significance threshold was set at *P* = .05, and 2-sided testing was used. Statistical analysis was performed using SPSS statistical software version 19 (IBM Corp) from June 2018 to May 2020.

## Results

A total of 74 participants were evaluated. Of those, only 68 participants (130 eyes) met the inclusion criteria and were evaluated in this study (median [interquartile range] age, 28 [24–36] years). Thirty-seven participants (54%) self-identified as male.

The mean (SEM) logarithm of the minimum angle of resolution (LogMAR) scores of both eyes together were 0.063 (0.014) for the treatment (smart speaker) and 0.049 (0.013) for the control (human) groups (*P* = .04). The mean (SEM) difference between the smart speaker and human LogMAR scores for both eyes together was 0.014 (0.001) (approximately 1 letter per examination) with a 95% CI of 0.001 to 0.029 and Cronbach α of 0.928. The reported precision and accuracy for both eyes (CCC, 0.8585; 95% CI, 0.7837 to 0.9088) were 0.8722 and 0.9843, respectively (Table). Bland-Altman analysis revealed a mean difference of −0.01 (95% CI, −0.13 to 0.10 ) LogMAR units between the human- and smart speaker–reported values for both eyes (Table).

## Discussion

To our knowledge, this study is the first to assess the feasibility of an autonomously administered visual acuity examination using a smart speaker device in a clinical setting. Although the differences in the reported data between Alexa and human values were statistically significant, in practice they were clinically insignificant and negligible at approximately 1 letter per examination. This study was limited by its small sample size. However, the results of this feasibility study are promising and suggest that the smart speaker performed comparably to the trained humans. With the growing adoption of telemedicine, smart speakers have the potential to be deployed in remote and low-resource settings for use by nonophthalmic personnel for screening and data collection.^4,5^ This could be explored in future studies.

Although some may question the utility of automating visual acuity examinations, this is an important first step toward the automation and validation of more complex screening examinations. Automation will become an increasingly necessary tool to augment and extend the reach of eye care practitioners as they adopt a more population-health management approach in the near future, especially in remote, low-resource, or other underserved settings.^5,6^

## Notes

Supplement. **eAppendix.** Supplemental Methods **eReferences.**
