# Supplementary material for: Feasibility of Use of a Smart Speaker to Administer Snellen Visual Acuity Examinations in a Clinical Setting
Source: JAMA Netw Open. 2020 Aug 19;3(8):e2013908. doi: 10.1001/jamanetworkopen.2020.13908 (PMC7439105; doi:10.1001/jamanetworkopen.2020.13908)
Supplement: Supplement. — eAppendix. Supplemental Methods eReferences. [file jamanetwopen-3-e2013908-s001.pdf]

Supplementary Online Content

Ismail HO, Moses AR, Tadrus M, Mohamed EA, Jones LS. Feasibility of use of a smart speaker to administer Snellen visual acuity examinations in a clinical setting. *JAMA Netw Open*. 2020;3(8):e2013908. doi:10.1001/jamanetworkopen.2020.13908

eAppendix. Supplemental Methods

eReferences

This supplementary material has been provided by the authors to give readers additional information about their work.

© 2020 Ismail HO et al. *JAMA Network Open.*

Supplemental Methods

Setting and Subject Recruitment

This non-randomized cross-over study was conducted at the Howard University (HU) Department of Ophthalmology from April 11, 2018 to May 18, 2018, and was approved by the HU Institutional Review Board. Participants were recruited through convenience sampling[1] at the Howard University College of Medicine and the HU Ophthalmology Clinic and included those who met the eligibility criteria. In order to be eligible, participants needed to be: a) at least 18 years of age, b) competent to give informed consent, c) able to read the Snellen chart, d) able to complete the exam, and e) not have speech impediments. Informed consent was required before enrollment, participation was voluntary, and no compensation was provided for participation.

Apparatus and Test Environment

All test procedures were conducted at the Howard University Department of Ophthalmology. Visual acuity for the control values were measured using a computerized Snellen chart Lombart CVS iMAC Acuity System version 3.9 running on a 20” iMac display at a distance of 2.44 meters from the patient and a height of approximately 1.27 meters. In addition, the visual acuity for the treatment values was measured in a different exam room using a combination of a second-generation Amazon Echo Dot approximately 0.3 meters from the patient and a custom computerized Snellen chart program displayed on an HP Spectre x360 convertible laptop in tablet mode with a 15.6” display at 3 meters from the patient and a height of 1.37 meters from the floor.

Testing Procedures

Each study participant served as their own control, and underwent two versions of the Snellen visual acuity exam: a) the standard computerized exam (the control group) administered by trained personnel (i.e., an ophthalmologist, resident, or medical student) and b) the autonomously administered exam by Alexa (the treatment group). In both versions of the exam, participants were instructed to wear any glasses or contacts they were prescribed in order to measure their best corrected visual acuity (BCVA) for their right eye (OD), left eye (OS), and both eyes (OU). Measurements were recorded as the BCVA of both eyes together or the BCVA of the only eye able to read the Snellen chart.

During the control measurements, participants were instructed to read the letters on the screen until they had difficulty identifying the letters. Visual acuity was recorded as the last line of which participants were able to read half the letters or more. For the treatment measurements, Alexa provided instructions and randomized the letters between exams to prevent participants from memorizing the charts. The proprietary Alexa skill was invoked for the eye being measured (i.e., “Alexa, launch right eye”), and Alexa would instruct the participants to cover the opposite eye and read the letters on the screen. Incorrect responses (i.e., wrong letters or pauses longer than eight seconds) would trigger Alexa to re-prompt the participants to read the letters on the

© 2020 Ismail HO et al. *JAMA Network Open.*

screen, giving the study participant another opportunity to read the letter correctly. A second incorrect response would be recognized as a true error, and the exam would proceed until the participant failed to read at least half of the letters on a line. At that point, the exam for that eye would be concluded and the visual acuity recorded. Once the exam for one eye was complete, the exam for the next eye was invoked, followed by a final invocation to test both eyes together. After completion of all visual acuity testing, all de-identified participant results of the control and treatment exams were then sent to a cloud database for later review.

Statistical Analysis

The visual acuity measurements for both the control and treatment groups were converted into logMAR units (Logarithm of the Minimum Angle of Resolution) for analysis. Data analysis was performed using SPSS statistical software version 19, (Chicago, IL). The median with interquartile ranges (IQR) was used to report non-normal distribution of quantitative data (i.e., age). A paired, two-tailed t-test compared the means, standard errors of means, and confidence intervals (CI). Reliability was assessed using tau-equivalent reliability (Cronbach's alpha). The concordance correlation coefficient (CCC) and Bland-Altman plot were used to assess agreement and reliability between the Alexa and human administered tests. The CCC reports both the precision (ρ, Pearson's correlation coefficient) and accuracy (C_β, bias correction factor) of the test in comparison to trained human examiners. With a sample size of 63 patients (126 eyes), a paired t-test with a 0.050 two-sided significance level will have 80% power to detect an effect size of 0.25 (i.e. a difference in means of 0.05 divided by 0.2 standard deviation) nQuery Advisory version 7.0 (Statistical Solutions Ltd., Cork, Ireland).

eReferences

1. Jager, J., D.L. Putnick, and M.H. Bornstein, *II. MORE THAN JUST CONVENIENT: THE SCIENTIFIC MERITS OF HOMOGENEOUS CONVENIENCE SAMPLES.* Monogr Soc Res Child Dev, 2017. **82**(2): p. 13-30.
2. O'Brien, R.G., Muller, K. E., *Unified Power Analysis for t-tests through Multivariate Hypotheses*, in *Applied Analysis of Variance in Behavioral Science* L. K. Edwards, Editor. 1993, Marcel Dekker: New York. p. 297-344.

© 2020 Ismail HO et al. *JAMA Network Open.*
